# Sensitive Voltammetric Sensor for Tryptophan Detection by Using Polyvinylpyrrolidone Functionalized Graphene/GCE

**DOI:** 10.3390/nano10010125

**Published:** 2020-01-09

**Authors:** Quanguo He, Jun Liu, Jinxia Feng, Yiyong Wu, Yaling Tian, Guangli Li, Dongchu Chen

**Affiliations:** 1 School of Materials Science and Energy Engineering, Foshan University, Foshan 528000, China; hequanguo@126.com; 2 School of Life Sciences and Chemistry, Hunan University of Technology, Zhuzhou 412007, China; fengjinxia0828@163.com (J.F.); wyy5082010@163.com (Y.W.); tianyaling0212@163.com (Y.T.); guangli010@hut.edu.cn (G.L.)

**Keywords:** polyvinylpyrrolidone, graphene, tryptophan, electrochemical behavior, voltammetric determination

## Abstract

In this paper, an electrochemical method for the measurement of tryptophan (Trp) was developed based on a glassy carbon electrode modified with polyvinylpyrrolidonefunctionalized graphene (PVP-GR)/glassy carbon electrode (GCE). In 0.1 M phosphate buffer solution (PBS, pH = 2.2), compared with bare GCE, PVP/GCE, and GR/GCE, the oxidation peak current of Trp increased dramatically at PVP-GR/GCE. The oxidation mechanism of Trp on the PVP-GR/GCE was discussed and the experimental conditions were optimized. Under the best experimental conditions, the oxidation peak current of Trp was proportional to its concentration in the range of 0.06 µM–10.0 µM and 10.0–100.0 µM, and the limit of detection (LOD) was 0.01 µM (S/N = 3). The target modified electrode with excellent repeatability, stability and selectivity, was successfully applied to detectTrp in drugs and biological samples.

## 1. Introduction

Tryptophan (Trp) is one of the essential amino acids in the human body which plays various roles. One of its important functions is as a precursor of many neurotransmitters and neurochemicals, including melatonin and serotonin. Serotonin is necessary to improve emotional and mental health and melatonin is known to help improve sleep. It is reported that there is a significant correlation betweenTrp concentration in plasmaand depression [1]. Trp is also required for normal growth in infants and nitrogen balance in adults. Unable tobe produced in the human body, Trp must be taken in from food or supplements. However, excessive Trp may produce a toxic waste in the brain, causing delusions and hallucinations [2]. In addition, it is thought to be a possible cause of schizophrenia in humans who cannot metabolize it properly [3,4,5]. Because of this clinical background, a convenient quantitative method for Trp is expected to bring many medical benefits.

Trp is usually determined by high-performance liquid chromatography [6], capillary electrophoresis [7], spectrophotometry [8], fluorescence [9] and chemiluminescence [10]. At the same time, Trp is an electroactive substance, which can be detected by the electrochemical method. Electroanalytical techniques have the advantages of simplicity, portability, selectivity, and sensitivity. It has great attraction for Trp monitoring in various sample matrices. However, direct oxidation of Trp on the bare electrode is not attractive because of slow electron transfer and high overpotential, as well as poor repeatability due to the fouling effect. In recent years, a lot of research work has been devoted to the introduction of new materials for electrode modification for Trp detection [11,12,13,14,15,16,17,18,19,20] (Table 1). However, there are still some disadvantages, such as low sensitivity or narrow linear range [14,17], highdetection limit [11,14], complexity [13,14,15], as well as the high cost of gold [12,17], will have an adverse effect on Trp detection. Particularly in biological fluids, uric acid (UA), ascorbic acid (AA), and dopamine (DA) often coexist, and their oxidation peak potentials always overlap, which results in poor selectivity for Trp detection. Therefore, it is still greatly important to develop new enhanced materials anddesign a novel sensor for Trp detection.

Graphene (GR) is a single layer composed of carbon atoms tightly arranged in a two-dimensional honeycomb lattice. Since the experimental discovery of monolayer by Novoselov and Geim [21], GR has attracted much attention due to its unique properties. The applications of GR or GR-based nanocomposites in high sensitivity and high selectivity electrochemical sensors have been widely reported, such as dopamine [22,23,24], 4-nitrophenol [25], rutin [26], tryptophan [27], sunset yellow [28], quinoline yellow [29], glucose [30], serotonin [31], acetaminophen [32], 4-aminophenol [33] and methyl parathion [34] sensors. Although GR has significant electrocatalytic and sensing properties, it has been reported that in most solvents, due to van der Waals interaction and strong π–π stacking, GR nanosheets are clustered together in a short time [35]. This will restrict its application in electrode modification. In order to overcome this disadvantage, in the past several strategies have been developed, such as the use of surfactants [36], ionic liquids [37], chitosan [38], or polymers with specific functions [39,40]. However, the desire to explore a better method to disperse GR in aqueous solutions has been increasing among the researchers.

Polyvinylpyrrolidone (PVP) is a non-toxic polymer and non-ionic surfactant. In the present work, the experimental results showed that PVP can effectively prevent the agglomeration of carbon atoms in GR nanosheets. Compared with pure GR, the dispersion and stability of polyvinylpyrrolidonefunctionalized graphene (PVP-GR) composite were significantly improved [41,42]. The PVP-GR composite was cast on the surface of a glassy carbon electrode (GCE) to obtain the modified electrode, which showed improved electrochemical response to Trp oxidation. The electrochemical behavior of Trp on PVP-GR/GCE was studied by calculating the electrochemical parameters. The sensor has been applied to the determination of Trp in drugs and biological samples [43] with satisfactory results. This study will expand the application of GR based materials in the field of electrochemical sensors.

## 2. Experimental

### 2.1. Chemical Agents and Solutions

Graphite, polyvinylpyrrolidone, 80 wt% hydrazine solution, 25 wt% ammonia solution, 30 wt% hydrogen peroxide solution were offered from Sinopharm Chemical Reagent Co., Ltd., Suzhou, China.The amino acids like Trp, ascorbic acid, uric acid, oxalic acid, lactic acid, tartaric acid, dopamine and glucose were obtained from a pharmaceutical company, Aladdin Chemical Reagent Co., Ltd., Shanghai, China. The compound amino acid injections were purchased from a pharmaceutical corporation called Xuzhou the Fifth Pharmaceutical Corporation, Xuzhou, China (17AA-I, Trp: 0.430 g L^−1^) and Guangzhou Green Cross Pharmaceutical Corporation located in Guangzhou, China (17AA-H, Trp: 0.700 g/L; 18AA-I, Trp: 1.00 g/L).The human blood serum samples were supported by the Hospital of the University of South China. A proper amount of Trp was dissolved and diluted to 100 mL to prepare 1.0 × 10^−3^ M Trp as the standard stock solution, which should bekept at 4 °C and can be stable for twoweeks. The analytical solution was newly prepared by diluting the Trp standard stock solution previously used. Unless otherwise specified, all chemicals reagents were of analytical grade and used as received. The double-distilled water was used to prepare all aqueous solutions.

### 2.2. Instruments

Cyclic voltammetry (CV) and chronocoulometry (CC) were implemented on a CHI 660E electrochemical workstation (Chenhua Instrument Co., Ltd., Shanghai, China) under the controlof CHI660 software in a microcomputer. The second-order derivative linear sweep (SDLSV) voltammograsms was recorded by a model JP-303E polarographic analyzer (Chengdu Instrument Factory, Chengdu, China) for electroanalytical measurements. A three-electrode system consisted of the bare or modified GCE (d = 4 mm), a platinum wire and a saturated calomel electrode (SCE), which worked as the working electrode, the counter electrodeand the reference electrode, respectively. All potentials were studied versus the SCE. pH was measured by a pH-3c Model pH meter (Shanghai Leichi Instrument Factory, Shanghai, China) with a combined glass electrode. Scanning electron microscope (EVO10, ZEISS, Jena, Germany) was used for the characterization test, and the images of it were obtained at 2.0 KV acceleration voltage.

### 2.3. Synthesis of GR and PVP-GR Composite

According to the previous report [19], graphite oxide was synthesized from natural graphite powder. Graphite oxide with the weight of 100 mg was dissolved in 100 mL of double distilled water and exfoliated to graphene oxide (GO) by ultrasonic treatment for 2 h. The resulting light yellow dispersion was subsequently centrifuged at 4000 rpm. 10.0 mg PVP was mixed with the homogenous GO dispersion (20.0 mL) in a flask, and the mixture was stirred at room temperature for 10 min, and then 20 µL hydrazine hydrate solution (50 wt%) and 80 µL ammonia solution (25 wt%) were added. After stirring vigorously for a few minutes, the flask was placed in an oil bath (95 °C) for 1 h. A stable black dispersion was obtained after the reaction, implying the reduction of GO. After the centrifuging and washing with ethanol and water in turn, the suspension was obtained and dried at room temperature. Finally, 1.0 mg PVP-GR composite was dispersed in 1.0 mL of water, and the uniform PVP-GR dispersion was preparedafter the ultrasonication for 5 min. As a control, GR suspension without PVP was prepared by the same method.

### 2.4. Electrode Preparation

Before modification, the GCE was polished to a mirror-like surface on a polishing cloth with 0.05 µm alumina slurries, sonicated in ethanol and double-distilled water, and dried under an infrared lamp [44]. For the preparation of PVP-GR/GCE, 10 µL of the PVP-GR dispersion was dropped on the pretreated GCE with a microsyringe, and dried under an infrared lamp. For comparison, PVP/GCE and GR/GCE were also prepared by casting 10 µL PVP solution (1.0 mg L^−1^) and 10 µL GR dispersion (1.0 mg mL^−1^) on the clean GCE surface by the similar procedures.

### 2.5. Electrochemical Measurements

The traditional three-electrode system was used to determine Trp in 10 mL electrochemical cells. Trp was determined in 0.1 M phosphate buffer (pH 2.2) unless otherwise indicated. The cyclic voltammograms (CVs) of Trp at PVP-GR/GCE were depicted in the range of 0.5~1.2 V at a scan rate of 0.1 V s ^−1^. The second derivative linear sweep voltammograms (SDLSVs) of Trp was plotted in the range of 0.3~1.1 V after stirring at 0.0 V for 60 s. The oxidation peak current measured at 0.890 V was used for the quantitative analysis of Trp. After each measurement, the modified electrode was scanned several times in the blank electrolyte to remove any adsorbate. All measurements were performed at room temperature.

## 3. Results and Discussion

### 3.1. Characterization of the Surface of GR/GCE and PVP-GR/GCE by SEM

**Figure 1:**
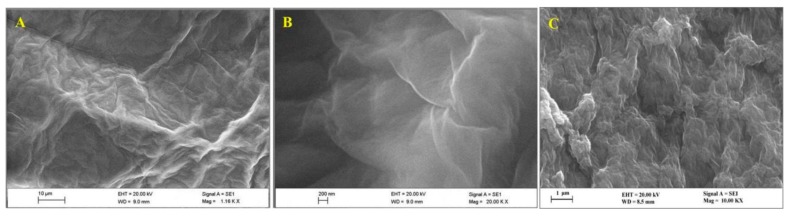
SEM pattern of GR (**A**,**B**) and PVP-GR composite (**C**). (Accelerating voltage: 20 kV, magnification times: 10,000).

The morphology of GR, PVP-GR composite modified on the GCE was observed by SEM (Figure 1). Figure 1A illustrates the typical SEM image of the prepared GR sheets. As shown in this figure, GR clearly shows the flake-like shapes. The high magnification SEM image of GR was shown in Figure 1B, clearly revealing the crumpled and wrinkled structure of GR on the electrode. These observations demonstrated that GR was successfully synthesized. The SEM image of the PVP-GR composite was exhibited in Figure 1C. It can be seen that a more wrinkled, more crumpled and layered structure was formed on the surface of GCE. Corrugation and rolling are the inherent characteristics of GR nanosheets. This winkled nature of GR is very beneficial to maintain a high specific surface area of the electrode. The above results show that PVP and GR are well combined, and the existence of PVP greatly prevents the aggregation of GR.

### 3.2. Electrochemical Characterization of Different Electrodes by CV

**Figure 2:**
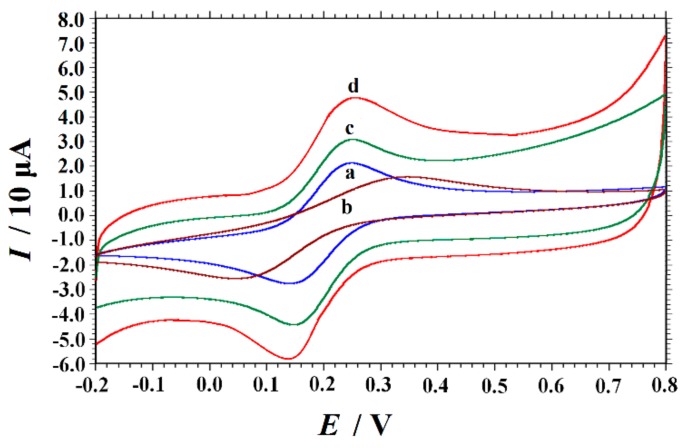
Cyclic voltammograms (CVs) of 1.0 mM potassium ferricyanide containing 1.0 M KCl solution plotted at different electrodes: (**a**) GCE, (**b**) PVP/GCE, (**c**) GR/GCE and (**d**) PVP-GR/GCE. Scan rate: 0.1 V s^−1^.

In addition, the electron transfer properties of the electrodes after different surface modifications were characterized by CV. Figure 2 showed the CVs of GCE, PVP/GCE, GR/GCE and PVP-GR/GCE in a 1.0 mM K_3_[Fe(CN)_6_] solution containing 1.0 M KCl at a scan rate of 0.1 V s^−1^. There are a couple of quasireversible redox peaks that appeared on bare GCE, and the peak-to-peak separation (Δ*E*_p_) is 109 mV. The oxidative peak current (*I*_pa_) and reduction peak current (*I*_pc_) were determined to be 24.03 µA and 24.16 µA (curve a). When PVP was coated on the surface of GCE, the redox peak current decreased and Δ*E*_p_ increased to 298 mV. This is due to the presence of PVP film on the electrode surface which prevents the diffusion of [Fe(CN)_6_]^3−/4−^ from solution to the electrode (curve b). Meanwhile, the redox peaks increased significantly (*I*_pa_ = 28.26 µA and *I*_pc_ = 32.43 µA) on the GR/GCE with the value of Δ*E*_p_ as 103 mV (curve c). This may be due to the good conductivity of GR, which increased the electron transfer rate of [Fe(CN)_6_]^3−/4−^ on the electrode surface. When PVP-GR/GCE was used, the redox peak current further increased (curve d). In addition, the background current of PVP-GR/GCE also increased, indicating that PVP-GR significantly increased the specific surface area of the electrode. According to the Randles–Sevcik equation [45]:

*I*_pc_ = (2.69 × 10^5^)*n*^3/2^*D*^1/2^*v*^1/2^*AC*, (1)

where *I*_pc_ is defined as the reduction peak current (*A*), *n* is defined as the number of electron transfer, *A* is defined as the effective surface area of the electrode (cm^2^), *D* is defined as the diffusion coefficient of K_3_[Fe(CN)_6_] in the solution (7.6 × 10^−6^ cm^2^ s^−1^[39]), *C* is defined as the concentration of K_3_[Fe(CN)_6_] (mol cm^−3^) and *v* is defined as the scan rate (V s^−1^). The effective surface area of GCE and PVP-GR/GCE was obtained as 0.1028 cm^2^ and 0.1791 cm^2^ by investigating the redox peak current with the scan rate. The results clearly demonstrated that the effective area of the electrode surface was greatly enlarged, thus greatly improving the performance of the modified electrode.

### 3.3. Electrochemical Behavior of Trp

**Figure 3:**
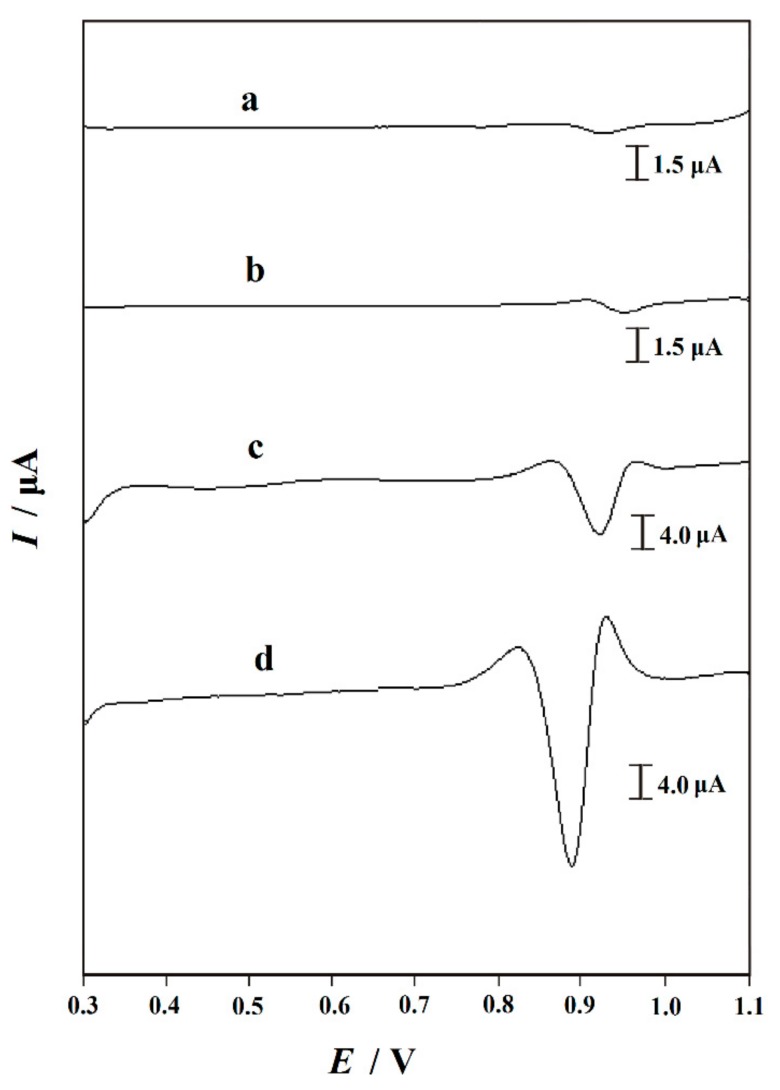
Second derivative linear sweep voltammograms (SDLSVs) of 10 µM tryptophan (Trp) in 0.1 M phosphate buffer (pH 2.2) recorded at (**a**) bare GCE; (**b**) PVP/GCE; (**c**) GR/GCE and (**d**) PVP-GR/GCE. Accumulation potential: 0.0 V, accumulation time: 60 s, scan rate: 0.1 V s^−1^.

The electrochemical behavior of Trp at different electrodes was studied by second derivative linear sweep voltammetry. Compared with CV, differential pulse voltammetry (DPV) and square wave voltammetry (SWV), SDLSV has the advantages of stable baseline and sharp peak shape, which helps to improve the sensitivity and selectivity of quantitative analysis [19,20]. Figure 3 exhibitsSDLSVs of 10 μM Trp obtained at different electrodes. As shown in Figure 3, after 60 s of deposition at 0.0 V, only a small and wide oxidation peakof 926 mV appeared on GCE, and the anodic peak current (*I*_pa_) was 0.4313 μA (curve a), indicating that the direct electron transfer of Trp was slow on bare GCE. When the surface of GCE was modified with PVP, *I*_pa_ increased slightly (0.5860 μA), which might be attributed to the hydrogen bonding interactions between PVP and Trp. When GR/GCE was used, the oxidation peak current of Trp at 908 mV increased to 8.713 µA. Compared with bare GCE and PVP/GCE, the peak current of Trp at GR/GCE increased significantly, and the peak potential shifted negatively, which was due to the good electrocatalytic performance and high conductivity of GR. Meanwhile, the peak current of Trp at 886 mV obtained on PVP-GR/GCE was the highest (28.29 µA). As we all know, preventing aggregation is particularly important for GR because most unique properties are only related toindividual form. The PVP-GR composite with excellent dispersed in water effectively expands the surface area of the electrode. On the other hand, due to the good electrocatalytic activity and conductivity of GR, the electron transfer rate of Trp on the electrode surface is greatly accelerated. Therefore, the synergistic effect of PVP and GR makes the peak current of Trp increase significantly, and greatly improves the sensitivity of determination.

**Figure 4:**
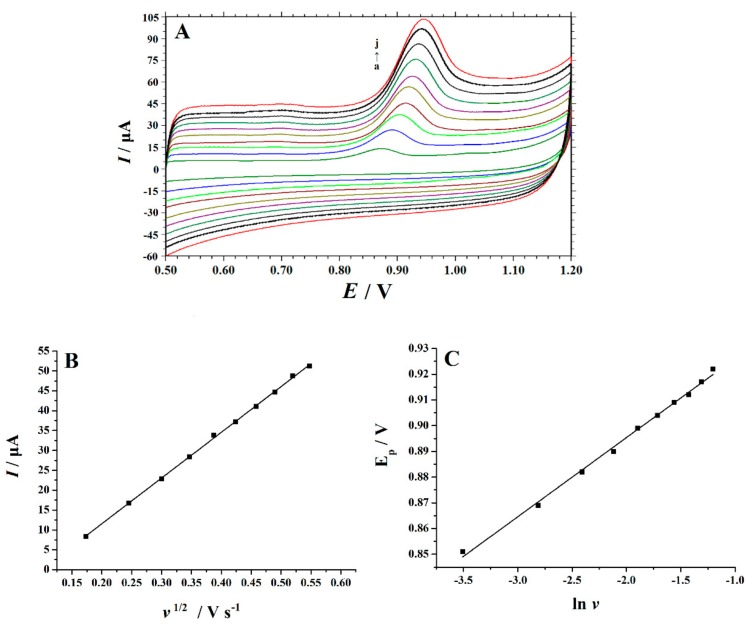
(**A**) CVs of 10 µM Trp at the PVP-GR/GCE in 0.1 M phosphate buffer solution (PBS) (pH 2.2) with different scan rates. a–g were 0.03, 0.06, 0.09, 0.12, 0.15, 0.18, 0.21, 0.24, 0.27, 0.30 V s^−1^, respectively; (**B**) the plot of oxidation peak currents (*I*_p_) vs. the square root of scan rates (*v*^1/2^); (**C**) the peak potentials (*E*_p_) vs. the Napierian logarithm of scan rates (ln*v*).

As shown in Figure 4, the effect of scan rate (*v*) on the oxidation of Trp was studied by cyclic voltammetry. At the rate of 0.03~0.3 V s^−1^,the peak current of Trp has a linear relationship with the square root of the scanning rate (*v*). The regression equation is *I*_p_ = 115.06 *v*^1/2^ − 11.459 (*I*_p:_ µA, *v*: V s^−1^) with the correlation coefficient (R^2^) of 0.9995. It showed that the oxidation of Trp on PVP-GR/GCE is a typical diffusion-controlled electrode process. In addition, it was observed that *E*_p_ was linearly correlated with ln*v*. The equation can be expressed as *E*_p_ = 0.0308 ln*v* + 0.957 (*E*_p_: V, *v*: V s^−1^), R^2^ = 0.9967. According to Laviron’s theory [40], the slope of the line is equal to *RT*/*αnF*, where *n* is the electron transfer number involved in rate-determining step, *α* is transfer coefficient. Therefore, it is easy to calculate the value of *αn* from the slope of *E*_p_ versus ln*v*. In this work, the slope is 0.0308, so *αn* is calculated as 0.8312. In general, *α* is assumed to be 0.5 in a completely irreversible electrode process. So, the electron transfer number (*n*) is 2.

### 3.4. Optimization of Experimental Parameters

#### 3.4.1. Effect of the Amount of PVP-GR Suspension

**Figure 5:**
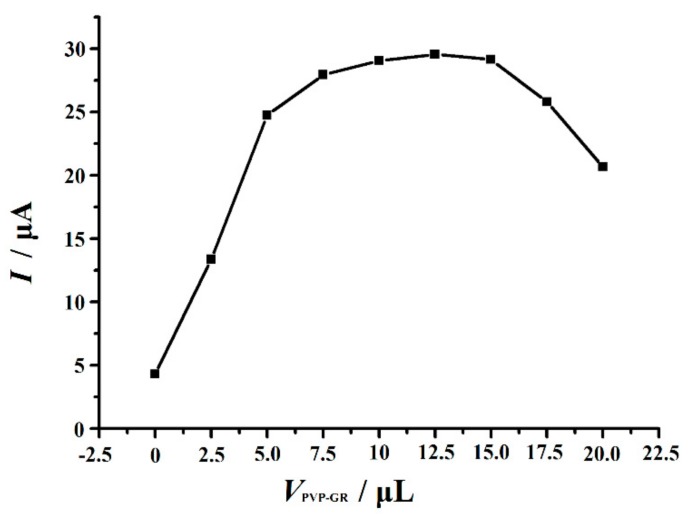
Effect of the amount of the PVP-GR suspension on the oxidation peak current of 10 µM Trp. Other conditions are the same as in Figure 3.

The effect of the amount of PVP-GR suspension of the electrode surface on the performance of the modified electrode was studied in the range of 0–20 μL, and the results were shown in Figure 5. It was found that in the range of 0–10 μL, the current signal of Trp enlarged with the increase of the volume of PVP-GR suspension fixed on the electrode. If the quantity of PVP GR suspension further added from 10 μL to 15 μL, the oxidation peak current of Trp can be basically considered to maintain invariable. However, when the amount of PVP-GR suspension was more than 15 μL, the oxidation peak current decreased, which may be attributed to the increase of the thickness of PVP-GR composite film, resulting in the increase of the interface electron transfer resistance, making the electron transfer more difficult. Moreover, with the increase of PVP-GR content, the background current increased, which makes it impossible to determine the trace level of Trp. Therefore, 10 μL PVP-GR suspension fixed on the electrode was selected for subsequent experiments.

#### 3.4.2. Effects of Supporting Electrolyte and Solution pH

The effect of different supporting electrolytes on the current response of Trp was studied by SDLSV. In basic and neutral electrolytes, such as NaNO_3_, KCl and NH_3_-NH_4_Cl (each 0.1 M), no or very weak current response was observed. However, in acidic buffer, such as phosphate buffer solution (PBS), HAc-NaAc buffer solution, HAc-NH_4_Ac buffer solution, sodium tartrate—tartaric acid buffer solution, (CH_2_)_6_N_4_-HCl solution, the current response of Trp was significantly enhanced. According to the peak height and peak shape, 0.1 MPBS was selected as the best supporting electrolyte.

**Figure 6:**
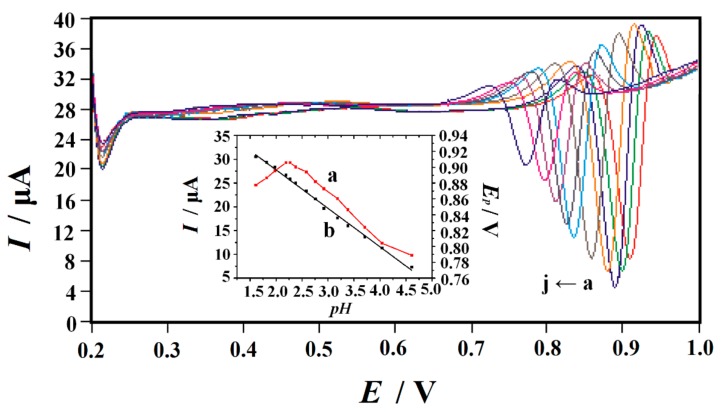
SDLSVs of 10 µM Trp at PVP-GR/GCE in 0.1 M PBS with different pH values a–j: 1.61, 1.98, 2.20, 2.38, 2.76, 3.18, 3.38, 3.71, 4.04 and 4.61, respectively. Inset: (**a**) the relationship between the peak current (*I*_p_) and pH; (**b**) the relationship between the peak potential (*E*_p_) and pH. Other conditions are the same as in Figure 3.

The pH value of the solution is another important factor affecting the electrochemical response of Trp. In the 10 µM Trp containing 0.1 MPBS with pH range from 1.6 to 4.6, the effect of pH on the current response was studied (Figure 6). With the enhancement of pH value from 1.6 to 2.2, it was obviously found thatthe oxidation peak current of Trp elevated gradually.While the pH exceeds 2.3, the oxidation peak current decreasedcontinuously. Previous studies have shown similar results [11,12]. Considering the sensitivity of Trp determination, pH 2.2 was chosen for the subsequent experiments. The relationship between the oxidation peak potential (*E*_p_) of Trp and the solution pH was also shown in Figure 6. With the enhancement of pH value, the linear shift of *E*_p_ to negative potential indicates that proton was directly involved in Trp oxidation, and it abided by the following equation: *E*_p_ (V) = −0.0486 pH + 0.9939 (R = 0.9982). The slope of −0.0486 V pH^−1^ indicated that the electron transfer is accompanied by the same number of proton transfer in the electrode reaction. In Section 3.3, we proved that the number of electron transfer in the oxidation process of Trp is two. Therefore, the electrooxidation of Trp on PVP-GR/GCE is a two-electron and two-proton process. This conclusion is undifferentiated from the known electrochemical reaction of Trp previously reported [11,14,15].

#### 3.4.3. Effect of Accumulation Potential and Time

**Figure 7:**
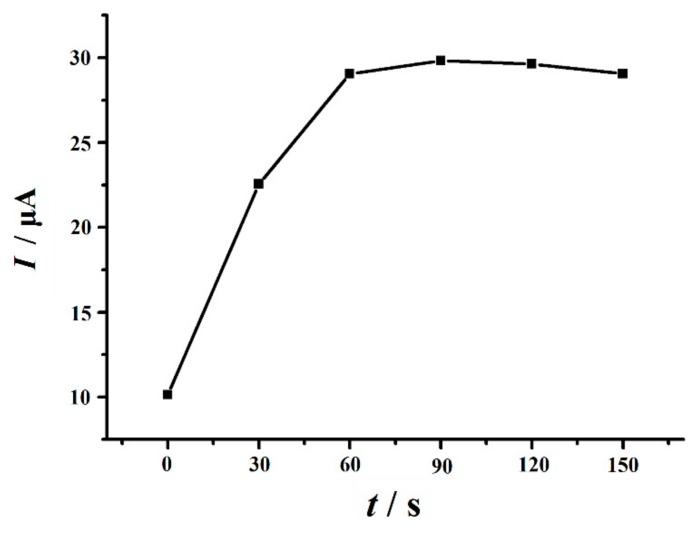
Effect of accumulation time on the peak current of 10 µM Trp in 0.1 M PBS (pH 2.2). Accumulation potential: 0.0 V, scan rate: 0.1 V s^−1^.

The effect of accumulation potential and accumulation time on the peak current of 10 µM Trp was studied. The peak current of Trp changed slightly when the accumulation potential changed from −0.3 to 0.3 V. According to the result from the experiments, the accumulation was carried out at 0.0 V as the optimal accumulation potential in this work. The effect of accumulation time on the peak current of Trp was also investigated (Figure 7). The result shows that the peak current increased rapidly untill the accumulation time reached 60 s. With the further increase of the accumulation time, however, the peak current decreased slightly. These indicated that Trp reached saturation rapidly on the surface of the modified electrode, and 60 s was used for accumulation for subsequent experiments.

### 3.5. Chronocoulometric Curve

**Figure 8:**
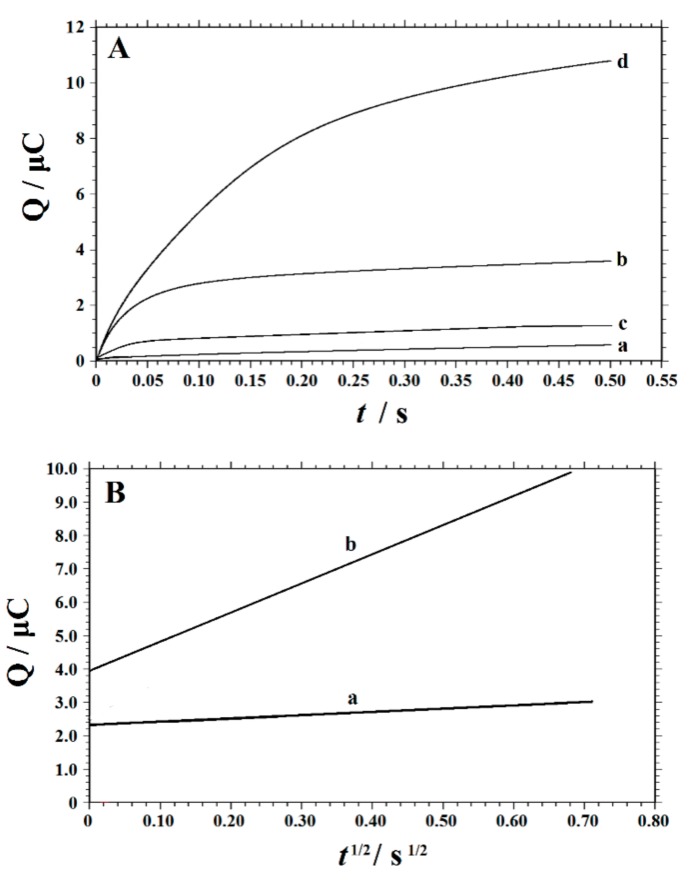
(**A**) Chronocoulometric curves in 0.1 M PBS (pH 2.2). Curve a and b for bare GCE in the absence and presence of 0.2 mM Trp, curve c and d for PVP-GR/GCE in the absence and presence of 0.2 mM Trp. (**B**) The plot of *Q*versus *t*^1/2^ at GCE (a) and PVP-GR/GCE (b) after background charge correction.

Since the electrode reaction was diffusion-controlled, the chronocoulometric response of Trp on the PVP-GR/GCE was investigated to calculate the diffusion coefficient (D) of Trp in the solution. Figure 8 showed the chronocoulometric curves obtained at the bare GCE and PVP-GR/GCE in the given solutions and the plot of net charge (point-by-point background subtraction) against *t*^1/2^ showed straight lines. The slope was gotten as 1.006 × 10^−6^ C s^−1/2^ for GCE and 8.170 × 10^−6^ C s^−1/2^ for PVP-GR/GCE, respectively. According to Anson’s equation [46]:

*Q* = 2*nFAc*D^1/2^π^−1/2^*t*^1/2^ + *Q_dl_ + Q_ads_*, (2)

where *n* is the number of electrons transferred, *F* (C mol^−^^1^) is the Faraday constant, *A*(cm^2^) is the effective surface area of working electrode, *c* (mol cm^−3^) is the concentration of substrate, *D* (cm^2^ s^−1^) is the diffusion coefficient, *Q_dl_* (C) is the double layer charge and *Q_ads_* (C) is the adsorption charge, other symbols have their usual significances. As *n* = 2, *c* = 2.0 × 10^−7^ mol cm^−3^, *A* = 0.1028 cm ^2^(GCE) and 0.1791 cm^2^ (PVP-GR/GCE) determined in Section 3.1, it was figured out *D* = 5.04 × 10^−8^ cm^2^ s^−1^ at GCE, which is increased to 1.10 × 10^−6^ cm^2^ s^−1^ at PVP-GR/GCE. All of these demonstratedthat the electrochemical reaction was accelerated on the PVP-GR/GCE on account of the presence of high conductive GR on the GCE surface.

### 3.6. Interference Study

**Figure 9:**
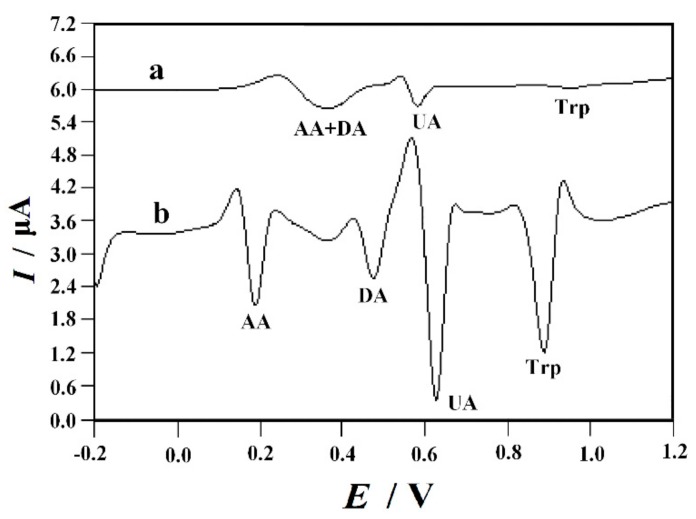
SDLSVs obtainedat the (**a**) bare GCE and (**b**) PVP-GR/GCE in the presence of 0.5 mM ascorbic acid (AA), 50 µM dopamine (DA), 10 µM uric acid (UA) and 10 µM Trp. Other conditions are the same as in Figure 3.

AA, UA and DA as three kinds of important biological substances often coexist with Trp in human fluid. Figure 9 exhibited the SDLSVs obtained at bare GCE and PVP-GR/GCE in the presence of 0.5 mM AA, 50 µM DA, 10 µM UA and 10 µM Trp. The results showed that four oxidation peaks were well separated in 0.1 M PBS (pH 2.2), the oxidation peak potentials of AA, DA, UA, and Trp were 0.196 V, 0.476 V, 0.628 V, and 0.886 V, respectively. It was found that there was no obvious interference for the oxidation signal of 10 µM Trp (signal change below 5%) in the presence of 100-fold concentrations of AA, 20-fold concentrations of DA and 10-fold concentrations of UA. The interference of other common coexisting substances was also investigated. The results showed that 1000 concentrations of Na^+^, K^+^, Mg^2+^, Cu^2+^, Ca^2+^, Al^3+^, Pb^2+^, Cl^−^, NO_3_^−^, SO_4_^2^^−^; 500-fold concentrations of oxalic acid, glucose, lactic acid, tartaric acid hardly interfere with the oxidation signal of10 µM Trp (signal change below 5%). Additionally, the interference effects of various amino acids were also explored. Most amino acids have no effect on the response signal of Trp except tyrosine (Tyr). The oxidation peak potential of Tyr is very close to Trp, which seriously interferes with the determination of Trp.

### 3.7. Repeatability, Reproducibility, and Stability

In order to evaluate the repeatability of the modified electrode, successive measurements were carried out in a 10 µM Trp solution at a single PVP-GR/GCE for seven times. The relative standard deviation (RSD) was 3.6%. In addition, the reproducibility of the PVP-GR/GCE was estimated by comparing the oxidation peak currents of 10 µM Trp obtained at seven different modified electrodes prepared by the same method. The RSD is 4.7%. The stability of the PVP-GR/GCE was estimated by measuring the response of 10 µM Trp within one month, and the electrode was stored in air at room temperature. It was found that the current response of 10 µM Trp at the electrode decreased to 94% after 10 days and remained 88% of the original response after 20 days. Even after 30 days, the electrode still maintained 83% of its original response.

### 3.8. Calibration Curve

**Figure 10:**
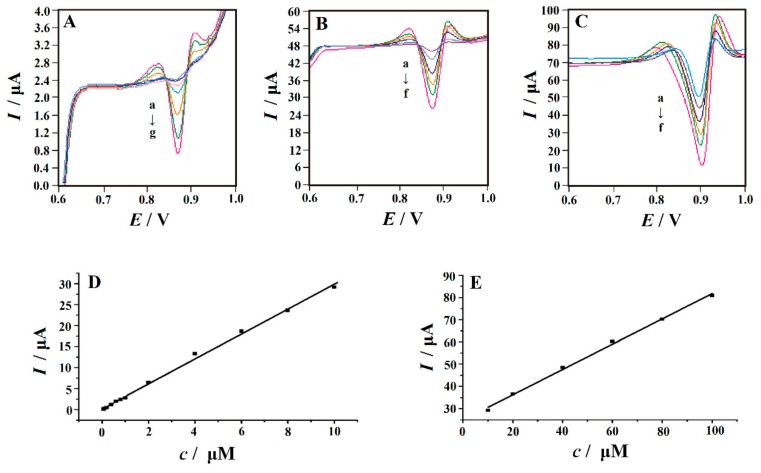
SDLSVs obtained at PVP-GR/GCE in 0.1 M PBS (pH 2.2) containing different concentrations of Trp. (**A**) from a to g is corresponding to 0.06, 0.08, 0.1, 0.2, 0.4, 0.6, 0.8 µM; (**B**) from a to f is corresponding to 1.0, 2.0, 4.0, 6.0, 8.0, 10.0 µM; (**C**) from a to f is corresponding to 10, 20, 40, 60, 80, 100 µM. The calibration curves for the determination of Trp in the concentration range of (**D**) 0.06–10 µM and (**E**) 10–100 µM, respectively. Other conditions are the same as in Figure 3.

The SDLSV was used for quantitative analysis, and the voltammograms of Trp with different concentrations were obtained as shown in Figure 10. Under the optimum conditions, the oxidation peak current of Trp increased linearly with its concentration in the range of 0.06 µM to 10 µM and 10 µM to 100 µM. The linear regression equation was *I* (µA) = 0.1554 + 2.9756*c* (µM) (r = 0.9987) and *I* (µA) = 24.845 + 0.5694*c* (µM) (r = 0.9987). The detection limit was calculated as 10 nM (S/N = 3). For apractical comparison with the previous methods, the comparative analysis figures of merit for various electrodes for the determination of Trp were given in Table 1. Obviously, the performance of the PVP-GR/GCE is comparable to or superior to the electrodes reported in terms of linear range and detection limit. In addition, the materials used in the electrode preparation are simple and low cost, making it a very attractive sensor in Trp analysis.

### 3.9. Sample Determination

The content of tryptophan in the compound amino acid injections were determined by this method. Firstly, the sample solution was diluted to 100-fold with redistilled water. Then 1.0 mL of the diluted solution was further diluted with 0.1 M phosphate buffer (pH 2.2), anddetermined under the optimum conditions.The standard addition method was used to evaluate the accuracy of the method. The results of four parallel measurements were showed in Table 2. It can be seen from Table 2 that the quantitative results of this electrochemical method are in good agreement with the data provided by the manufacturer, and the recovery was between 97.0% and 104.0%, indicating that PVP-GR/GCE can be effectively applied to the detection of Trp in commercial pharmaceutical samples. This method was also applied to the biological samples such as human serum and urine to prove the practical applications. The preparation of the serum samples wasbased onthe previous report [47,48,49,50]. The samples were diluted with PBS and the standard Trpsolution was supplemented to calculate the recovery. The results were shown in Table 3. It can beobserved that an acceptable recovery of 96.0–103.6% was obtained, and illustrate that PVP-GR/GCE could be applied for Trp determination in the biological samples.

## 4. Conclusions

A new electrochemical method for the determination of Trp based on the PVP-GR/GCE was proposed. Graphene prepared by chemical reduction in the presence of PVP has good stability and dispersion in aqueous solution. PVP-GR composite greatly improves the specific surface area and conductivity of the electrode, and the sensor has high catalytic activity in promoting electron transfer of Trp. The results showed that this method has a wide linear range, low detection limit, high selectivity, good stability and excellent reproducibility for Trp detection. In particular, this method has been successfully used to determine the concentration of Trp in drugs and biological samples, and can be used for routine analysis of Trp in clinical use.

## Figures and Tables

**Table 1:** Comparison of the efficiency of polyvinylpyrrolidonefunctionalized graphene (PVP-GR)/glassy carbon electrode (GCE) with other modified electrodes in the electrochemical determination of tryptophan.

Electrode	Technique	Supporting Electrolyte	Linear Range/µM	Detection Limit/µM	References
^a^ 4-ABA/GCE	^k^ LSV	phosphate buffer (pH 2.0)	1.0–100	0.2	[11]
^b^ Au-NPs/GCE	^l^ DPV	phosphate buffer (pH 2.5)	0.09–50	0.08	[12]
^c^ SnO_2_-Co_3_O_4_@rGO/IL/CPE	DPV	B-R buffer (pH 3.0)	0.02 to 6.00	0.0032	[13]
^d^ BuCh/GCE	DPV	phosphate buffer (pH 7.0)	2–60	0.6	[14]
^e^ Ag-MoS_2_/CS/GCE	DPV	phosphate buffer (pH 6.0)	0.5–120	0.05	[15]
^f^ ß-CD/MWCNTs/GCE	DPV	phosphate buffer (pH 3.0)	1.5–30.5	0.07	[16]
^g^ EGPU-tAuNP	DPV	Britton-Robinson buffer (pH 7.4)	0.6–2.0	0.053	[17]
^h^ rGO/SnO_2_/GCE	DPV	phosphate buffer (pH 7.0)	1–100	0.04	[18]
^i^ ErGO/ABPE	second derivative LSV	0.1 M H_2_SO_4_	0.1–10; 10–100	0.06	[19]
^j^ MIP/ABPE	second derivative LSV	phosphate buffer (pH 7.0)	0.01–4; 4–20; 20–100	0.008	[20]
PVP-GR/GCE	second derivative LSV	phosphate buffer (pH 2.2)	0.06–10 and 10–100	0.01	This work

^a^ 4-aminobenzoic acid polymer film modified glassy carbon electrode; ^b^ gold nanoparticles modified glassy carbon electrode; ^c^ reduced graphene oxides decorated with SnO_2_-Co_3_O_4_ nanoparticles modified ionic liquid carbon paste electrode; ^d^ butyrylcholine modified glassy carbon electrode; ^e^ silver nanoflakes-molybdenum sulfide/chitosan modified glassy carbon electrode; ^f^ β-cyclodextrin incorporated with multi-walled carbon nanotubes modified glassy carbon electrode; ^g^ gold nanoparticles modified graphitepolyurethane composite electrode; ^h^ reduced graphene oxide decorated with tin oxide nanoparticles modified glassy carbon electrode; ^i^ electrochemical reduced graphene modified acetylene black paste electrode; ^j^ molecular imprinted polymer modified acetylene black paste electrode; ^k^ linear sweep voltammetry; ^l^ differential pulse voltammetry.

**Table 2:** Detection of Trp in the compound amino acid injection samples (*n* ^a^ = 4).

Sample	Specified/µM	Detected ^b^/µM	RSD/%	Added/µM	Total Found ^b^/µM	Recovery/%
17AA-I ^c^	2.10	2.24	2.3	2.0	4.18	97.0
17AA-H ^c^	3.43	3.38	2.1	3.0	6.47	103.0
18AA-I ^c^	4.90	5.03	2.6	5.0	10.23	104.0

^a^ Thenumber of parallel measurements. ^b^ Average of four parallel measurements. ^c^ Dilution factor: 1/1000.

**Table 3:** Detection of Trp in the human serum and urine samples (*n* ^a^= 4).

Sample	Detected ^b^/µM	RSD/%	Added/µM	Total Found ^b^/µM	Recovery/%
Serum-1	2.79	2.9	3.0	5.84	101.7
Serum-2	3.85	3.2	4.0	7.98	103.2
Urine-1	Not detected	-	1.0	0.96	96.0
Urine-2	Not detected	-	5.0	5.18	103.6

^a^ The number of parallel measurements. ^b^ Average of four parallel measurements.
